# Mitonuclear discordance modulates mitochondrial ageing dynamics in natural *Drosophila* populations

**DOI:** 10.1073/pnas.2529208123

**Published:** 2026-07-07

**Authors:** Stefano Bettinazzi, Avishikta Chakraborty, Finley Grover-Thomas, Damian K. Dowling, M. Florencia Camus

**Affiliations:** ^a^ Department of Genetics, Evolution and Environment, University College London, London WC1E 6BT, United Kingdom; ^b^ School of Biological Sciences, Monash University, Melbourne, Victoria 3800, Australia

**Keywords:** mitonuclear discordance, mitohormesis, mitochondrial metabolism, ageing, *Drosophila*

## Abstract

Mitochondrial decline is a hallmark of ageing, yet the role of intergenomic compatibility in shaping ageing trajectories remains poorly understood, particularly in an ecologically relevant framework. Hormetic interventions have been proposed as strategies to modulate metabolism and lifespan, but it is unknown how this operates in the context of mitonuclear discordance. Here, we demonstrate that mitonuclear mismatch accelerates age-related mitochondrial decline, elevates reactive oxygen species production, and shortens lifespan. Strikingly, early-life mitochondrial stress induced by dietary modulation counteracts these effects, promoting mitochondrial homeostasis and longevity. Our findings reveal mitonuclear interactions shaping ageing trajectories in natural populations and provide unique evidence that targeted interventions can act as a buffer against the detrimental impact of genetic discordance.

Mitochondria lie at the center of cellular metabolism and are key determinants of organismal ageing (1). Because the oxidative phosphorylation (OXPHOS) complexes are encoded by both nuclear and mitochondrial genomes, compatibility between these genomes is essential for efficient energy production and eukaryotic life (2). Disruption of this intergenomic coordination, via mismatches between mitonuclear genotypes, has been shown to impair metabolism with severe life-history consequences across diverse taxa (3, 4). Yet, the role of mitonuclear compatibility in shaping ageing trajectories in natural populations remains poorly understood, with evidence largely limited to inbred laboratory lines (5, 6).

Hormesis describes the process where mild stress can trigger protective adaptations against ensuing perturbations (7). In this context, mitohormetic interventions can represent a protective strategy to promote metabolic homeostasis and healthy ageing (7, 8). Here, we leveraged natural genetic variation in wild *Drosophila melanogaster* populations to test how mitonuclear compatibility interacts with early-life metabolic stress to shape ageing phenotypes. Two mitochondrial haplotypes coexist in *D. melanogaster* populations along the Australian cline: “t” (most common in the north) and “m” (most common in the south), differing by 15 SNPs across protein-coding genes (9). We generated a panel of outbred populations carrying putatively coevolved (“tT,” “mM”) and mismatched (“mT,” “tM”) mitonuclear genomes (Fig. 1*A* and *SI Appendix*).

**Fig. 1.**
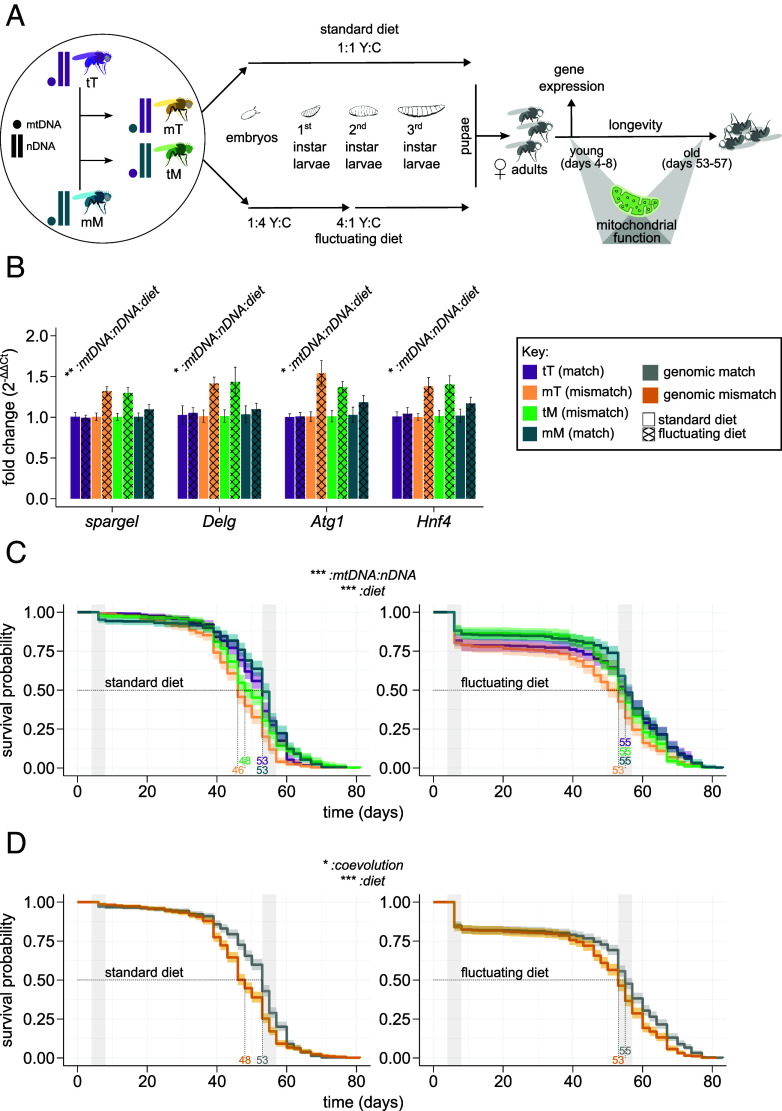
Mitonuclear and dietary interventions modulate gene expression and lifespan. (*A*) Experimental design. (*B*) Transcript abundance across mitonuclear populations and diets (mean ± EM) (*n* = 6 per group). (*C*) Survival by mitonuclear populations (*n* = 328 to 395 per group). (*D*) Survival by coevolutionary status (*n* = 718 to 787 per group). Kaplan–Meier curves with 95% CI; dashed lines: median survival; shaded areas: mitochondrial physiology assay windows. *P ≤ 0.05; **P ≤ 0.01; ***P ≤ 0.001.

Hormetic responses were explored by applying early-life metabolic stress through tailored isocaloric dietary modulation. We minimized the negative effects of dietary imbalance (10) by using macronutrient compositions shown to only cause moderate stress, without reducing egg-to-adult survival (11). Larvae were developed either under standard nutritional conditions (1:1 yeast-to-carbohydrate ratio; Y:C), or under fluctuating diet, where macronutrient availability was manipulated at key developmental stages (10). This involved an initial protein restriction phase (high-sugar diet – 1:4 Y:C) for 48 h, where larvae experienced reduced development, followed by high-protein exposure (4:1 Y:C) until adulthood to induce compensatory growth (*SI Appendix*, Table S1). We then measured gene expression of mitochondrial stress-related markers (*SI Appendix*, Table S2), adult lifespan, and mitochondrial function in young and old flies (O_2_ and reactive oxygen species—ROS fluxes) (Fig. 1*A* and *SI Appendix*).

## Results and Discussion

Gene expression analyses revealed strong dietary effects, with overall upregulation under fluctuating diet (*SI Appendix*, Table S3). This included genes involved in heat shock response (*Hsp22*), mitochondrial biogenesis (*spargel*, *Delg*), autophagy/mitophagy (*Atg1*, *Pink1*, *parkin*), and regulatory factors (*dSir2*, *dSirt4*, and *Hnf4*); consistent with mitochondrial stress induction (12). Notably, a subset of genes (*spargel*, *Delg*, *Atg1*, *Hnf4*) exhibited significant mitonuclear × diet interactions, showing cybrid-specific upregulation under fluctuating diet (Fig. 1*B* and *SI Appendix*, Table S3).

**Fig. 2.**
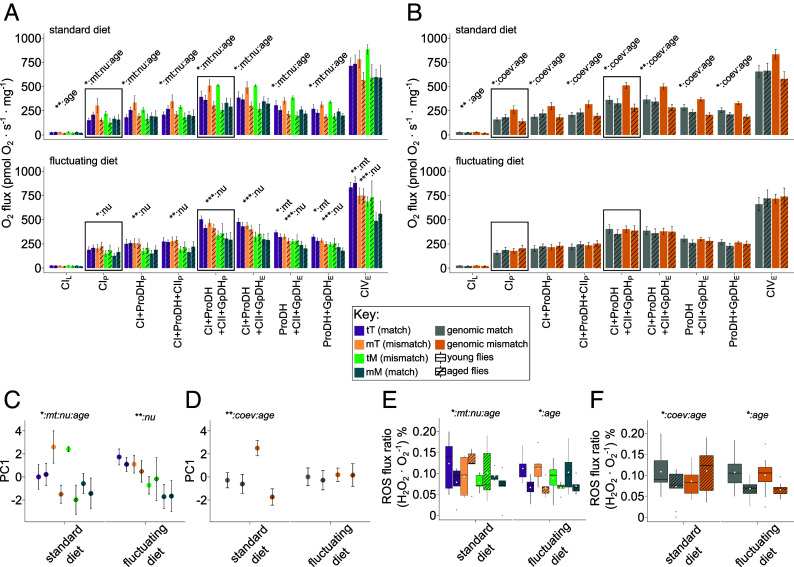
Mitonuclear and dietary interventions shape mitochondrial ageing. (*A* and *B*) Oxygen fluxes (pmol O_2_ • s^−1^ • mg^−1^) across respiratory states: _L_, leak; _P_, coupled; _E_, uncoupled. Respiration sustained by different combinations of respiratory complexes: complex I (CI), proline dehydrogenase (ProDH), complex II (CII), glycerophosphate dehydrogenase (GpDH), and cytochrome *c* oxidase alone (CIV). CI-linked respiration (CI_P_) and maximal coupled respiration (CI+ProDH+CII+GpDH_P_) are highlighted in boxes. (*C* and *D*) Principal component 1 (76% of the variability). (*E* and *F*) ROS flux ratio [(H_2_O_2_ • O_2_^−1^)%] during maximal respiration. Data (*A*−*D*) are mean ± SEM. mt: mtDNA; nu: nDNA; coev: coevolution status. *P ≤ 0.05; **P ≤ 0.01; ***P ≤ 0.001.

Under standard diet conditions, mitonuclear mismatch reduced median lifespan compared to coevolved populations (“coevolution” χ^2^ = 4.65, *P* = 0.03; Fig. 1 *C* and *D* and *SI Appendix*, Table S4). The reduced lifespan does not reflect a classic longevity–fecundity trade-off, as these cybrid populations have also shown reduced fertility in our previous work (3). At young age, mismatched lines exhibited higher respiratory rates than matched populations (Fig. 2 *A*–*D*). Consistent with our previous work, we interpret this increase as compensatory metabolic upregulation arising from intergenomic incompatibility, which is associated with reduced performance and deleterious life-history phenotypes in these lines (3, 11). Accordingly, respiratory function declined steeply with age in mismatched lines, with significant mitonuclear × age interactions across respirometry parameters (Fig. 2 *A* and *B* and *SI Appendix*, Table S5), including CI-linked respiration (CI_P_, F_1,32_ = 6.41, *P* = 0.016), maximal coupled respiration (CI+ProDH+CII+GpDH_P_, F_1,32_ = 5.99, *P* = 0.02), and principal component 1 (PC1, F_1,32_ = 6.95, *P* = 0.012), a composite measure of mitochondrial respiratory capacity (Fig. 2 *C* and *D*). Given the additive effects of the individual substrates, complex I appears to be the driver of the observed mitonuclear impact on respiration (Fig. 2 *A* and *B*). Additionally, ROS production during maximal respiration (integrating contributions from both mitonuclear- and nuclear-encoded complexes) increased only in aged-mismatched populations (F_1,24_ = 5.43, *P* = 0.028; Fig. 2 *E* and *F*). Grouping by mitonuclear match confirmed that declines in mitochondrial performance and lifespan were specific to mismatched combinations (Figs. 1 and 2 and *SI Appendix*, Tables S4, S5).

In contrast, moderate juvenile metabolic stress in the form of fluctuating diets had long-lasting beneficial effects on ageing patterns. Despite early survival costs, lifespan across all lines was extended compared to controls raised in standard conditions (“diet” X^2^ = 142.67, *P* < 0.001; Fig. 1 *C* and *D* and *SI Appendix*, Table S4). Here, respiration rates were driven by the nuclear background (all coupled and uncoupled fluxes, *P* < 0.03) (Fig. 2 *A* and *B* and *SI Appendix*, Table S5), and ROS production by age (F_1,7.8_ = 10.34, *P* = 0.012) (Fig. 2 *E* and *F* and *SI Appendix*, Table S5), rather than a mitonuclear combination. In sum, developmental intervention triggered the upregulation of genes linked to mitochondrial stress-response pathways, promoted mitochondrial homeostasis with age, and extended lifespan across all mitonuclear lines. These results are consistent with the concept of mitohormesis (7), and support the hypothesis that low-level metabolic stress can promote adaptive adjustments (7, 8, 12).

We further show that such buffering extends to populations exhibiting signatures of mitonuclear mismatch, indicating that diet-induced plasticity can mitigate genomic discordance. Notably, a subset of genes linked with mitochondrial dynamics and function (*spargel*, *Delg*, *Atg1*, *Hnf4*) (13–15) showed cybrid-specific upregulation, suggesting a prominent role for these pathways in promoting mitonuclear homeostasis. Future work should focus on the mitochondrial pathways identified here to elucidate the molecular mechanisms that underpin these responses. Further investigation in other genetic systems and with alternative stressors is also warranted to assess the generality of these effects beyond the specific context examined here.

This study shows that mitonuclear epistasis represents one biologically relevant context in which intergenomic compatibility shapes mitochondrial performance and ageing in natural populations. Moreover, our results indicate that early-life dietary manipulation has the potential to buffer age-related mitochondrial decline and extend lifespan in mismatched genotypes, consistent with hormetic responses. Together, these findings highlight how developmental plasticity can mitigate the physiological consequences of genomic discordance.

## Materials and Methods

We used a panel of four *Drosophila* populations with matched (tT, mM) and mismatched (mT, tM) mitonuclear combinations. Each population was subjected to a developmental dietary treatment: standard and fluctuating diet and mated upon eclosion. Gene expression in young females was quantified by qRT-PCR. Female lifespan was recorded and mitochondrial function (oxygen and ROS fluxes) at young (4 to 8 d) and old (53 to 57 d) age measured via High-Resolution FluoRespirometry. Detailed methods are provided in the *SI Appendix*.

## Supplementary Material

Appendix 01 (PDF)

## Data Availability

Datasets, Scripts, Stats data have been deposited in Figshare (16). Study data are included in the article and/or *SI Appendix*.
