# Supplementary material for: Mitonuclear discordance modulates mitochondrial ageing dynamics in natural Drosophila populations
Source: Proc Natl Acad Sci U S A. 2026 Jul 7;123(28):e2529208123. doi: 10.1073/pnas.2529208123 (PMC13367786; doi:10.1073/pnas.2529208123)
Supplement: Supplementary file 1 — Appendix 01 (PDF) [file pnas.2529208123.sapp.pdf]

SUPPORTING INFORMATION

Brief Report

Mitonuclear discordance modulates mitochondrial ageing dynamics in natural *Drosophila* populations

Stefano Bettinazzi[a*], Avishikta Chakraborty[a], Finley Grover-Thomas[a], Damian K. Dowling[b], M. Florencia Camus[a*]

[a] Department of Genetics, Evolution and Environment, University College London, London, UK [b] School of Biological Sciences, Monash University, Melbourne, Australia

* Corresponding authors: Stefano Bettinazzi; email: s.bettinazzi@ucl.ac.uk; M. Florencia Camus; email: f.camus@ucl.ac.uk

EXTENDED METHODS

Lines establishment and maintenance

Drosophila were sourced from a field collection that took place between December 2020 and March 2021, from cline-end wild populations in northern and southern Australia, which exhibit genomic signatures of local adaptation (1-3). Specifically, flies were collected from Townsville (latitude: -19.26, longitude 146.81) and Melbourne (latitude: -37.77, longitude: 144.99), and were designated accordingly as 'T' and 'M'. Mass-bred lines carrying northern ('T') or southern ('M') nuclear genomic background were established at Monash University (Australia) using virgin flies (1:1 sex-ratio) derived from 26-35 isofemale lines. To eliminate the cytoplasmic endosymbiont *Wolbachia*, flies were treated with tetracycline ($0.3\ mg \cdot mL^{-1}$), and its absence was confirmed via PCR amplification (2). Along the Australian east coast, *Drosophila* populations have been found to harbour two predominant mitochondrial haplotypes, which were found to vary in frequency across a latitudinal gradient in a 2012 collection. Northern subtropical populations were shown to predominantly carry the A1 haplotype (which we denote 't' in this paper), whereas the 'B1' haplotype (called 'm' here) is predominant in southern temperate populations. The two mtDNA haplotypes differ by 15 synonymous SNPs, which span most protein-coding genes (2). Starting from two cline-end populations (Townsville – north; Melbourne – south) and sourcing both mtDNA haplotypes from another set of strains

that were derived from a central location (Coffs Harbour), we used an established balancer chromosome crossing scheme (4, 5) to generate a full-factorial mitonuclear fly panel (see Supplementary figure 1 from Bettinazzi et al.(2024) for full crossing scheme figure (5)). The panel used a two-letter naming system, where the first letter (lowercase) represents the mitochondrial genome, and the second letter (uppercase) denotes the nuclear background. Among the four resulting populations, two carried putatively coevolved mitonuclear genomes reflecting the predominant mitonuclear combination in northern ('tT') and southern ('mM') populations (2). The remaining two populations had putatively mismatched mitonuclear genomes, where mitochondria with high frequencies in either north or south populations were placed alongside the nuclear background of the opposite cline-end population (i.e. 'mT' and 'tM'). Previous work on this system has shown that populations with matched mitonuclear combinations outperform mismatched populations for key life-history and physiological traits, supporting functional coadaptation between mitochondrial and nuclear genotypes, despite the mtDNA variants not being fixed at the cline ends (5, 6). All populations were reared at standard constant conditions (25°C, 50% RH, 1:1 yeast:carbohydrate Y:C diet, 12h:12h light:dark daily cycle). Nuclear genetic variability was preserved by regular backcrossing with wild cline-end paternal populations.

We regularly checked mtDNA genomic sequences across all populations to avoid possible contamination. Genomic DNA was extracted using the DNeasy Blood & Tissue Kit (Qiagen), quantified with a NanoDrop 2000 spectrophotometer (Thermo Fisher Scientific Inc), and finally set up for polymerase chain reaction on a Veriti thermocycler (AB Applied Biosystems) using a Phusion® High-Fidelity DNA Polymerase kit (New England BioLabs® Inc). To discriminate between 't' and 'm' haplotypes, we used a primer set (clineF: 5' *ACCTTTACGAAATTCCCATCCTCT* 3' and clineR: 5' *ATGATGCACCGTTAGCATGT* 3') which amplifies a ~200 bp region of the *cox2* gene carrying two haplotype-specific SNPs (2). PCR conditions were set as follow: 1X HF Buffer, 1.5 mM $MgCl_2$, 200 µM DNTPs, 0.4 µM of primers forward and reverse, 1U Phusion DNA Polymerase, and 100 ng DNA sample. Initial denaturation was carried out at 95°C for 120 s, and was followed by 30 cycles constituting of 30 s at 95°C for, 30 s at 60°C, and 60 s at 72°C. A final extension at 72°C for 7 min was performed. Amplifications were verified on 1% agarose gels, purified using the Wizard SV Gel and PCR Clean-Up System (Promega), sequenced (Sanger sequencing - Source Biosciences), and finally examined using Geneious Prime 2024.0.7 software.

Nutritional treatments and experimental set up

Putative hormetic responses were triggered by introducing moderate mitochondrial stress early in life using specifically designed dietary adjustments. In the generation prior to the longevity experiment, parental flies (parents to the assayed focal flies) from all four mitonuclear populations were left to oviposit for 16 hours on ovipositing plates with food media from two diet treatments. Our control treatment was standard (sugar–yeast agar medium with a yeast-to-carbohydrate ratio (Y:C) of 1:1), and our secondary treatment was a high-carbohydrate (1:4 Y:C) media; wherein the amount of yeast is a proxy for the amount of protein present in the diets (noting that the amount of proteins in the yeast used here is 40% of the total macronutrient concentration). From each oviposition plate, we picked ~40 eggs and placed them in treatment-matched food vials. The control group developed under standard nutritional conditions, whereas our treatment group (fluctuating diet – 'fd') underwent a food-swap after 48 hrs (2nd instar larvae). This intervention involved shifting larvae from the high-carbohydrate treatment to vials containing high-protein food media (4:1 Y:C). Increased protein in this treatment accelerated growth of the larvae that were developing slower than controls in the high-carbohydrate food. The diet shift was performed by floating the larvae in a 15% sucrose solution (7), gently collecting them with a mounted needle and lightly rinsing them in 1 x PBS solution. Finally, larvae were transferred into new vials containing the high-protein food and left to develop until adulthood. While the initial protein-deficient diet slowed larval development during the first 48-hour period, the subsequent high-protein diet accelerated growth, resulting in the simultaneous eclosion of both the control and treatment groups. Although we did not test fly body size after larval development, thorax weight (mg) of young flies did not differ across lines nor diets (linear mixed model - lmer(log(var) ~ mtDNA × nDNA × diet + (1|day); mean ± sem: tT-st = 1.04 ± 0.04; tT-fd = 0.95 ± 0.061; mT-st = 0.98 ± 0.058; mT-fd = 1.029 ± 0.061; tM-st = 1.02 ± 0.037; tM-fd = 1.086 ± 0.086; mM-st = 1.14 ± 0.068; mM-fd = 1.0 ± 0.053 - Table S5), giving an indication that larval dietary treatments might not impact body size in young adults. Each dietary treatment is isocaloric, and was previously describe to only cause mild stress (8). Recipes are reported in Table S1.

Gene expression quantification

Five day old mated female flies were used for transcript abundance analysis. A pool of five females was used for each sample, and a total of 6 samples per line and treatment (*n* =48) was flash-frozen in liquid nitrogen. Flies were homogenized in TRIzol™ Reagent (Invitrogen) using a pestle (20 strokes). Following addition of chloroform, the RNA-containing aqueous

phase was collected, and RNA extracted using the RNeasy Plus Mini Kit (Qiagen). RNA samples were stored at −80°C until required.

Reverse transcription was carried out with the RevertAid First strand cDNA synthesis KIT (ThermoFisher) using 1µg of total RNA and a 50/50 mixture of Oligo dT and Random Hexamer primers. The cycle consisted in 5 minutes incubation at 25°C, followed by 60 minutes at 42°C and 5 min at 70°C. Resultant cDNA samples were diluted to 100 ng · μL^{-1} and stored at −20°C until RT-qPCR analysis.

Transcript abundance was quantified for the following genes, associated with heat shock response (*Hsp22*, *Hsp60C*), mitochondrial biogenesis (*spargel*, *Delg*), autophagy/mitophagy (*Atg1*, *Pink1*, *parkin*), and regulatory factors (*dSir2*, *dSirt4*, *Hnf4*) (9, 10). Primers are reported in Table S2. Five reference genes were tested, including *EF1α*, *rp49*, *Act88F*, *RpoII*, and *rosy*. Primer specificity was confirmed with Primer Blast online tool (NCBI).

Gene expression was measured using quantitative real time (qRT)-PCR (Eppendorf Mastercycler Realplex^2). Reactions were performed in duplicate using a KAPA SYBR® FAST qPCR Master Mix (2x) Kit (KAPABIOSYSTEMS). cDNA template (2.5 µL) was incubated with 11.25 µL KAPA Buffer (1X), 0.45 µL of each primer (200 nM each), and H_2O (total volume: 22.5 µL). The amplification regime consisted of an initial 3 min at 95°C, 40 cycles of 5 s at 95°C, 10 s at 60°C, and 10 s at 72°C, followed by a final 1 min at 72°C.

The stability of the reference genes (Ct values) was assessed with RefFinder online tool (11). Four suitable genes were chosen: *EF1α*, *rp49*, *RpoII* and *rosy*, and the reference Ct (REF-Ct) was obtained from their geometric mean. Stability of the reference (REF-Ct) was confirmed by absence of effect (linear model - mtDNA * nDNA * diet), which indicated no significant differences among lines nor diets.

The expression of the target genes was expressed as ΔCt values (ΔCt = GOI-Ct – REF-Ct), calculated using the Ct values of the gene of interest (GOI) and the geometric mean of the four house keeping genes (REF-Ct). ΔΔCt values were obtained using line-specific calibrator group (line-specific average ΔCt value at control condition - standard diet). Fold change was calculated as $2^{-\Delta\Delta Ct}$ (Table S3).

Longevity

To minimise potential inter-vial variation, newly eclosed flies for each mitonuclear population were independently mixed in a common cage. Flies were let to acclimate and mate for 72 hours at standard conditions (25°C and standard food media), prior to setting up the lifespan assay. Four-day-old flies were anaesthetized using light CO_2 and sorted by sex. Females were

set up in vials containing standard food (1:1 Y:C) at a density of 20 flies per vial. A total of 17-20 vials were set up for each genotype-by-diet group (four genotypes, two dietary regimes); 3,140 individuals in total. Three times a week and for the entire duration of the study, female flies were transferred to new standard food, and number of deaths per vial recorded (Table S4). Flies that escaped during the transfer, or that were stuck in the media were censored from the study (12).

Mitochondrial function

Mitochondrial functionality was assessed on female flies undergoing the longevity assay at two different time windows – at young (4 to 8 days-old flies) and older age (53 to 57 days-old flies). Oxygen and reactive oxygen species (ROS) fluxes were screened through High-Resolution FluoRespirometry using two dedicated O2K machines (Oroboros Instruments Inc, Innsbruck, AS). Fly thoraces were dissected with tweezers on an ice-cold BIOPS preservation solution (2.77 mM CaK_2EGTA, 7.23 mM K_2EGTA, 5.77 mM Na_2ATP, 6.56 mM $MgCl_2$, 20 mM taurine, 15 mM Na_2phosphocreatine, 20 mM imidazole, 0.5 mM dithiothreitol, and 50 mM K-MES, pH 7.1) (13), and each sample was composed of two thoraces (14). Tissues were then permeabilized for 20 minutes on a saponin-BIOPS solution (80 $\mu g \cdot mL^{-1}$), rinsed in respiratory buffer for 5 minutes, dried, and weighted. Samples were finally transferred to O2K respiratory chambers prefilled with 2.1 mL of Mir05 respiratory buffer (110 mM D-sucrose, 60 mM lactobionic acid, 20 mM taurine, 20 mM HEPES, 10 mM KH_2PO_4, 3 mM $MgCl_2$, 0.5 mM EGTA, BSA 1 $g \cdot L^{-1}$) (13), and set to a temperature of 25°C. Oxygen signals were calibrated to air saturation, whereas fluorescence signals through stepwise titration of an H_2O_2 solution (0.1 µM each step), in presence of DTPA (15 µM), superoxide dismutase (SOD - 5 $U \cdot mL^{-1}$), horseradish peroxidase (HRP - 1 $U \cdot mL^{-1}$), and Amplex Ultra Red (AmR - 10 µM). Oxygen and ROS fluxes were then measured simultaneously while performing a personalized substrate-uncoupler-inhibitors-titration (SUIT) protocol (5, 14). The SUIT protocol first measured complex I (CI)-linked respiration sustained by the substrates pyruvate (P, 10 mM) and malate (M, 2 mM) in two respiratory states – i.e. LEAK (CI_L– state 4, in absence of ADP) and OXPHOS (CI_P - state 3, in presence of 5 mM ADP). It was followed by stepwise addition of substrates proline (Pro 10 mM), succinate (S, 10 mM) and glycerophosphate (Gp, 10 mM), to measure the respiratory contribution of proline dehydrogenase ($CI+Pro_P$), complex II ($CI+ProDH+CII_P$) and glycerophosphate dehydrogenase ($CI+ProDH+CII+GpDH_P$) complexes. Parameter $CI+ProDH+CII+GpDH_P$ also refers to the maximal coupled respiration achieved in the assay (respiration coupled with ATP production). Maximal uncoupled respiration (ETS-state, state 3u) $CI+ProDH+CII+GpDH_E$ was promoted by stepwise titration of FCCP (0.125 µM each step). Sequential addition of the inhibitors rotenone (Rot, 0.5 µM) and malonate (Mal, 5 mM) allowed

the measurement of respiration without the contribution of complex I (ProDH+CII+GpDH$_E$), and complexes I + II (ProDH+GpDH$_E$). Residual respiration (ROX) was measured following the addition of complex III-inhibitor antimycin A (Ama, 2.5 μM). Finally, concomitant addition of ascorbate (As, 2 mM) and TMPD (Tm, 0.5 mM) allowed the measurement of complex IV standalone activity (CIV$_E$), corrected for the residual oxygen flux obtained after addition of the complex IV inhibitor azide (Azd, 20 mM). H_2O_2 fluxes during max coupled respiration, as well as the maximal ROS production when OXPHOS was completely inhibited were also measured alongside respiratory rates. Respirometry data were expressed as oxygen (pmol $O_2 \cdot s^{-1} \cdot mg^{-1}$) and hydrogen peroxide (pmol $H_2O_2 \cdot s^{-1} \cdot mg^{-1}$) fluxes, normalized for tissue mass. Reactive oxygen species production rates were also expressed as ratios over the simultaneous step-specific oxygen consumption (($H_2O_2 \cdot O_2^{-1}$)%) (Table S5).

Statistical analyses

Statistical analyses were carried out using the R software (15). For longevity, 'survival' (16) and 'coxme' (17) packages were used. Kaplan-Meier (KM) models were implemented to estimate median lifespan and plot survival curves. A Cox Proportional Hazard Model was implemented to test fixed and interaction effects. The full model contained survival (time and type of the event) as a response variable, and considered factors 'mtDNA' (2 levels, northern 't' and southern 'm' mitochondrial genotypes), 'nDNA' (2 levels, northern 'T' and southern 'M' nuclear genotypes), and 'diet' (2 levels, standard 'st' and fluctuating 'fd' dietary treatments) as fixed effects, as well as two- and three-way interactions between those factors. Additionally, the impact of intergenomic coevolution (2 levels, coevolved 'coev' vs mismatched ´mism´ genomes) and 'diet' on survival, plus their interaction effect was specifically tested in a separate Cox Proportional Hazard Mixed Model, which also included factor 'strain' (4 levels, 'tT', 'mT', 'tM'. 'mM') as a random effect. Starting from a full model, the best fitting model was determined through step-wise simplification. The random effect structure was reduced in cases of singularity. Significance was determined by means of a Wald Chi-sq test.

For gene expression data, statistical analyses were performed on ΔCt values, while fold change ($2^{-\Delta\Delta Ct}$) was used for plotting (Table S3). Linear models considered ΔCt values as the response variable, and factors 'mtDNA', 'nDNA', and 'diet' as fixed effects, plus their interactions. The impact of mitonuclear coevolution status 'coev' (matched vs mismatched genomes) was formally tested in separate linear mixed models, also considering its interaction with factor 'diet'. Factor 'strain' was included as a random effect (Table S3).

For respirometry data, oxygen flux parameters were analysed separately, as well as condensed in principal components via principal component analysis ('FactoMineR' (18)

package). The first component (PC1) accounted for 76% of the total variability, with maximal uncoupled and coupled respiration ($CI+ProDH+CII+GpDH_E$ and $CI+ProDH+CII+GpDH_P$) contributing to it (13.98% and 13.82%, respectively), as well as $ProDH+CII+GpDH_E$ (12.46%), $ProDH+GpDH_E$ (12.29%), $CI+ProDH+CII_P$ (12.02%), CIV_E (11.36%), $CI+ProDH_P$ (11.04%), CI_P (10.62%), and CI_L (2.42%). The second component (PC2) accounted for 13.1% of the variability, with non-phosphorylating LEAK respiration (CI_L) highly contributing to it (39.20%), followed by $CI+ProDH_P$ (15.78%), CI_P (14.48%), $CI+ProDH+CII_P$ (11.94%), $ProDH+GpDH_E$ (8.45%), $ProDH+CII+GpDH_E$ (7.82%), CIV_E (1.91%), $CI+ProDH+CII+GpDH_E$ (0.38%), and $CI+ProDH+CII+GpDH_P$ (0.05%). These principal components (PC1 and PC2) were added to the respirometry dataset as additional parameters, together with single-step respiratory fluxes. For each parameter, linear mixed models were implemented using 'lme4' (19) and 'lmerTest' (20) packages. Models considered oxygen or ROS fluxes as the response variables, and factors 'mtDNA', 'nDNA', 'age', and 'diet' as fixed effects, plus their interactions. Factor 'day' (assay age, 10 levels, days 4–8 and days 53–57) was included as a random effect. The impact of mitonuclear coevolution status 'coev' (matched vs mismatched genomes) was formally tested in separate models, also considering its interaction with factors 'age' and 'diet'. Factors 'day' and 'strain' were included as random effects. Although higher variance is expected in the coevolved–mismatched analysis because two mitonuclear genotypes are pooled per category, this approach complements the full analysis by revealing general patterns in the data. Linear mixed models were also run separately for each dietary treatment ('st' and 'fd').

The best fitting model was determined through step-wise simplification of the full model. The random effect structure was reduced if singularity was detected. Significance was determined using Type III ANOVA (for models including interactions) or Type II ANOVA (for models without interactions), using Satterthwaite approximation of degrees of freedom. Assumptions were verified graphically and with the use of the 'performance' package (21). Statistical significance was set at $p \leq 0.05$. Effect sizes (partial η^2) were estimated using the package 'effectsize' (22), and represent how much variance associates with a factor or interaction in the model by accounting for the variance linked with the other variables. The effects were considered either small (0.01-0.05), medium (0.06-0.13), or large (≥0.14). Data and statistical analyses are reported in full in supplementary Tables S3,S4, and S5.

REFERENCES

1. A. A. Hoffmann, A. R. Weeks, Climatic selection on genes and traits after a 100 year-old invasion: a critical look at the temperate-tropical clines in Drosophila melanogaster from eastern Australia. *Genetica* **129**, 133-147 (2007).
2. M. F. Camus, J. N. Wolff, C. M. Sgro, D. K. Dowling, Experimental Support That Natural Selection Has Shaped the Latitudinal Distribution of Mitochondrial Haplotypes in Australian *Drosophila melanogaster*. *Molecular Biology and Evolution* **34**, 2600-2612 (2017).
3. Z. Lajbner, R. Pnini, M. F. Camus, J. Miller, D. K. Dowling, Experimental evidence that thermal selection shapes mitochondrial genome evolution. *Sci Rep* **8**, 9500 (2018).
4. D. J. Clancy, Variation in mitochondrial genotype has substantial lifespan effects which may be modulated by nuclear background. *Aging cell* **7**, 795-804 (2008).
5. S. Bettinazzi *et al.*, Assessing the role of mitonuclear interactions on mitochondrial function and organismal fitness in natural *Drosophila* populations. *Evolution Letters* **8**, 916-926 (2024).
6. S. Bettinazzi *et al.*, Mitonuclear interactions and early-life diet shape adult nutritional behaviour. *Journal of Evolutionary Biology* 10.1093/jeb/voaf123, voaf123 (2025).
7. M. Balakrishnan, W. J. Sisso, M. K. Baylies, Analyzing muscle structure and function throughout the larval instars in live Drosophila. *STAR Protocols* **2**, 100291 (2021).
8. S. Bettinazzi *et al.*, Mitonuclear interactions and early-life diet shape adult nutritional behaviour. *Journal of Evolutionary Biology* 10.1093/jeb/voaf123 (2025).
9. F. Hunter-Manseau, J. Cormier, N. Pichaud, From molecular to physiological responses: improved stress tolerance and longevity in *Drosophila melanogaster* under fluctuating thermal regimes. *Journal of Experimental Biology* **228** (2025).
10. Y. Hu *et al.*, FlyPrimerBank: an online database for Drosophila melanogaster gene expression analysis and knockdown evaluation of RNAi reagents. *G3 (Bethesda, Md.)* **3**, 1607-1616 (2013).
11. F. Xie, J. Wang, B. Zhang, RefFinder: a web-based tool for comprehensively analyzing and identifying reference genes. *Functional & Integrative Genomics* **23**, 125 (2023).
12. M. F. Camus, M. O'Leary, M. Reuter, N. Lane, Impact of mitonuclear interactions on life-history responses to diet. *Philosophical Transactions of the Royal Society B: Biological Sciences* **375**, 20190416 (2020).
13. E. Gnaiger, *Mitochondrial Pathways and Respiratory Control An Introduction to OXPHOS Analysis, 5th Edition* (Bioenergetics Communications, 2020), doi:10.26124/bec:2020-0002.
14. E. Rodríguez, S. Bettinazzi, S. Inwongwan, M. F. Camus, N. Lane, Harmonising protocols to measure *Drosophila* respiratory function in mitochondrial preparations. *Bioenergetics Communications* **2023.3** (2023).
15. R Core Team, R: A language and environment for statistical computing. *R Foundation for Statistical Computing, Vienna, Austria* (2021).
16. T. M. Therneau, P. M. Grambsch, *Modeling Survival Data: Extending the Cox Model* (Springer, New York, 2000).
17. T. Therneau, coxme: Mixed Effects Cox Models. R package version 2.2-22. 2024. (2024).
18. S. Lê, J. Josse, F. Husson, FactoMineR: An R Package for Multivariate Analysis. *Journal of Statistical Software* **25**, 1 - 18 (2008).
19. D. Bates, M. Mächler, B. Bolker, S. Walker, Fitting Linear Mixed-Effects Models Using lme4. *Journal of Statistical Software* **67**, 1 - 48 (2015).
20. A. Kuznetsova, P. B. Brockhoff, R. H. B. Christensen, lmerTest Package: Tests in Linear Mixed Effects Models. *Journal of Statistical Software* **82**, 1 - 26 (2017).
21. D. B.-S. Lüdecke, M.S. , I. Patil, P. Waggoner, D. Makowski, performance: An R Package for Assessment, Comparison and Testing of Statistical Models. *Journal of Open Source Software* **6**, 3139 (2021).

22. M. Ben-Shachar, D. Lüdecke, D. Makowski, effectsize: Estimation of Effect Size Indices and Standardized Parameters. *Journal of Open Source Software* **5**, 2815 (2020).
